# Brain Organoids as Model Systems for Genetic Neurodevelopmental Disorders

**DOI:** 10.3389/fcell.2020.590119

**Published:** 2020-10-12

**Authors:** Simona Baldassari, Ilaria Musante, Michele Iacomino, Federico Zara, Vincenzo Salpietro, Paolo Scudieri

**Affiliations:** ^1^Medical Genetics Unit, IRCSS Giannina Gaslini Institute, Genoa, Italy; ^2^Department of Neurosciences, Rehabilitation, Ophthalmology, Genetics, Maternal and Child Health (DiNOGMI), University of Genoa, Genoa, Italy; ^3^Pediatric Neurology and Muscular Diseases Unit, IRCSS Giannina Gaslini Institute, Genoa, Italy; ^4^Department of Neuromuscular Diseases, UCL Queen Square Institute of Neurology, London, United Kingdom

**Keywords:** brain organoids, *in vitro* models, stem cells, 3D-culture, neurodevelopmental disorders, autism spectrum disorders, epilepsy

## Abstract

Neurodevelopmental disorders (NDDs) are a group of disorders in which the development of the central nervous system (CNS) is disturbed, resulting in different neurological and neuropsychiatric features, such as impaired motor function, learning, language or non-verbal communication. Frequent comorbidities include epilepsy and movement disorders. Advances in DNA sequencing technologies revealed identifiable genetic causes in an increasingly large proportion of NDDs, highlighting the need of experimental approaches to investigate the defective genes and the molecular pathways implicated in abnormal brain development. However, targeted approaches to investigate specific molecular defects and their implications in human brain dysfunction are prevented by limited access to patient-derived brain tissues. In this context, advances of both stem cell technologies and genome editing strategies during the last decade led to the generation of three-dimensional (3D) *in vitro*-models of cerebral organoids, holding the potential to recapitulate precise stages of human brain development with the aim of personalized diagnostic and therapeutic approaches. Recent progresses allowed to generate 3D-structures of both neuronal and non-neuronal cell types and develop either whole-brain or region-specific cerebral organoids in order to investigate *in vitro* key brain developmental processes, such as neuronal cell morphogenesis, migration and connectivity. In this review, we summarized emerging methodological approaches in the field of brain organoid technologies and their application to dissect disease mechanisms underlying an array of pediatric brain developmental disorders, with a particular focus on autism spectrum disorders (ASDs) and epileptic encephalopathies.

## Introduction

Neurodevelopmental disorders (NDDs) encompass a range of frequently co-existing conditions that include intellectual disability (ID), developmental delay (DD), and autism spectrum disorders (ASDs) (Heyne et al., 2018; Salpietro et al., 2019). ASDs represent a complex set of behaviorally defined phenotypes, characterized by impairments in social interaction, communication and restricted or stereotyped behaviors (Chen et al., 2018). Epilepsy and NDDs frequently occur together, and when refractory seizures are accompanied by cognitive slowing or regression, patients are considered to have an epileptic encephalopathy (EE) (Scheffer et al., 2017). Both ID and ASDs are clinically and etiologically heterogeneous and a unifying pathophysiology has not yet been identified for either the disorder as a whole or its core behavioral components (Myers et al., 2020). Family and twin studies suggest high (0.65–0.91) heritability (Chen et al., 2018) and genetic dissection of the complex molecular architecture of ID/ASD is revealing contributions from both coding and non-coding DNA changes (Williams et al., 2019). Chromosomal microarray and next-generation sequencing (NGS) led over the last decade to the identification of a number of *de novo* and inherited variants implicated in the molecular etiology of ID/ASD variably associated with epilepsy (Wang et al., 2019). Deleterious variants in the same genes are often implicated in multiple NDDs characterized by autistic features and other comorbidities such ID, seizures or developmental epileptic encephalopathies, and neuropsychiatric conditions including schizophrenia and attention-deficit/hyperactivity disorder (O’Donovan and Owen, 2016). Defining the full spectrum of defective molecular pathways will help diagnose, monitor and accelerate treatment development in genetic NDDs (Lombardi et al., 2015). Currently, susceptibility and major mendelian alleles identified in NDDs explain only a small percentage of risk, and most of the work is still ahead to uncover the complex genetics of these disorders.

Also, assessing the pathophysiological mechanism(s) underlying brain developmental disorders is historically challenging due to limitations in accessing human brain and the complexity of the central nervous system (CNS). Studies on animal models, particularly mice, gained insight to various genetic and environmental conditions impacting neurodevelopment and neuronal functions, but rarely achieved to recapitulate precisely the most complex human neurological phenotypes. This could be attributed to human brain complexity and size with many features that are species-specific; as instance, the human cerebral cortex is over 1.000 times larger in terms of area and number of neurons (Leung and Jia, 2016) and extensive differences exist between homologous brain regions in humans and mice, including marked alterations in cell types proportions, distributions, morphology, and gene expression (Hodge et al., 2019). These features unique to humans highlight the importance of investigating the human brain by accurate models capable to recapitulate its development at different stages.

Several recent advances in biotechnology, including stem cells culture and cell reprogramming methods, CRISPR-Cas9-based genome editing, biomaterials, optogenetics, and single-cell transcriptomics, allowed the generation and the fine characterization of complex 3D-models, i.e., the cerebral (or brain) organoids. These *in vitro* 3D-models, with respect to rodent models, may facilitate investigation of complex neurodevelopmental human diseases by combining different important determinants of the human features. In particular, brain organoids generated from human pluripotent stem cells (hPSC) preserve the human genomic context, allowing to study the pathogenetic mechanisms of diseases associated with monogenic as well as polygenic genomic alterations. Moreover, brain organoids recapitulates species-specific developmental timing *in vitro*, such as cell cycle dynamics, duration of neurogenic period, rate and pattern of cell migration (Mariani et al., 2015; Toma et al., 2016). Brain organoids represent also an obvious advance with respect to 2D cell cultures, enabling studying more complex phenotypes involving different neuronal networks, tissue architecture, and organ morphogenesis.

In this review, we focused on the most recent advances in cerebral organoids technology, and on its application to the study of genetic NDDs, particularly ASDs and epilepsy.

## Brain Organoids and Technologies

Brain organoids are self-organized 3D-aggregates generated from hPSC or induced pluripotent stem cells (iPSC) with cell types and cytoarchitectures resembling the embryonic human brain (Eiraku et al., 2008; Kadoshima et al., 2013; Lancaster et al., 2013; Paşca et al., 2015). Multiple brain organoid protocols and techniques have been exploited in the last 5–10 years, ranging from the development of region-specific organoids to more complex whole-brain organoids that recapitulate cell interactions and interconnectivity between multiple brain regions (Lancaster and Knoblich, 2014a,b; Paşca, 2018; Xiang et al., 2020a).

**FIGURE 1:**
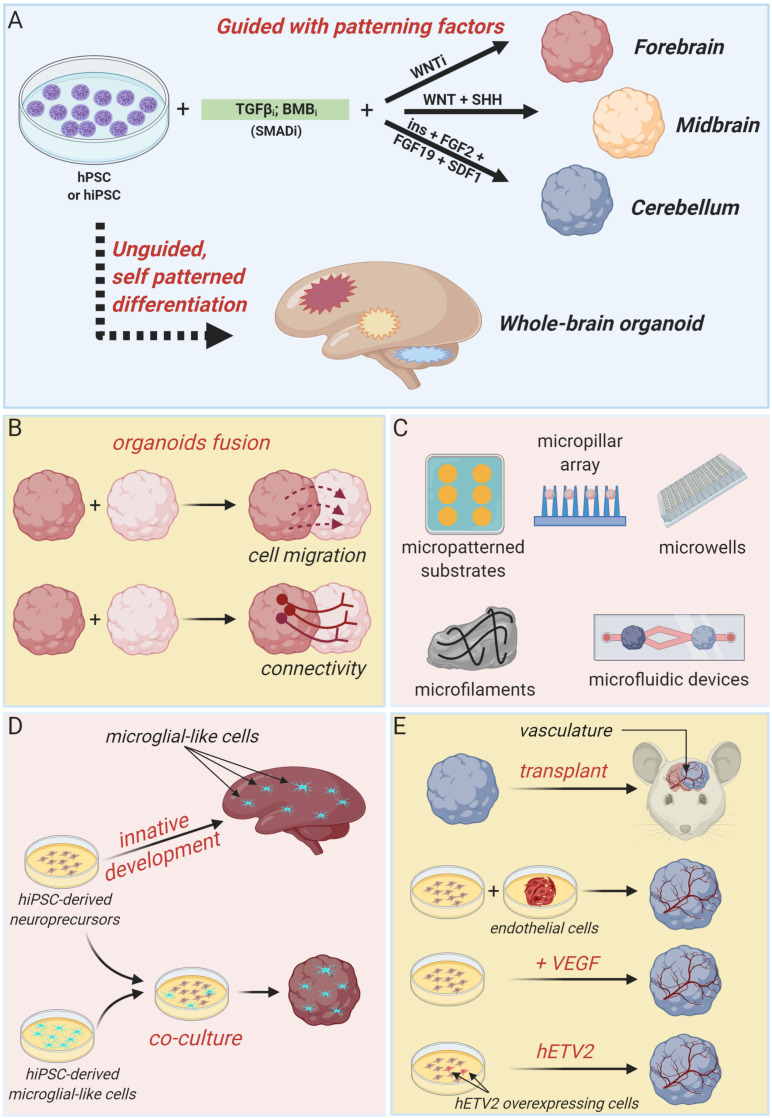
Brain organoid technologies. **(A)** Schematic summary of guided and unguided methods to generate different types of cerebral organoids. Guided approaches allow the generation of region-specific brain organoids resembling discrete parts of the developing human brain, such as forebrain, midbrain or cerebellum. Unguided approaches, instead, result in the generation of organoids resembling the whole-human brain. **(B)** Region-specific organoids can be fused to each other to model regional-interactions, such as cellular migration and long-range connectivity. **(C)** Summary of bioengineering tools to regulate brain organoids morphology and structure. **(D)** Cartoon illustrating two independent strategies to develop microglia-containing brain organoids. Microglial-like cells can innately develop within unguided-whole brain organoids or can be introduce by co-culture methods. **(E)** Different attempts to obtain organoid vascularization, including organoids transplantation in nude mice, co-culture with iPSC-derived endothelial cell precursors and co-differentiation of neuronal and endothelial cells by VEGF administration or hETV2 overexpression (created with biorender.com).

Generally, approaches to generate brain organoids can be categorized in two major classes: unguided and guided methods (Figure 1A). Unguided methods rely on spontaneous morphogenesis and intrinsic differentiation capacities of hPSC aggregates (Lancaster et al., 2013; Renner et al., 2017). In the original protocol developed by the Knoblich’s lab, embryoid bodies derived from hPSC aggregates are embedded in droplets of Matrigel (to mimic extracellular matrix) and then transferred to a spinning bioreactor to enhance nutrient absorption and, thus, facilitate tissue expansion and neural differentiation (Lancaster et al., 2013; Lancaster and Knoblich, 2014b). With minimal external influence, this method produces large (up to 2–4 mm diameter) and heterogeneous cerebral tissue displaying discrete, but interdependent, brain regions (forebrain, midbrain, and hindbrain) and subregions (various cortical lobes, choroid plexus, and retina) (Lancaster et al., 2013). Single-cell transcriptomic approaches confirmed the heterogeneous cellular population of unguided cerebral organoids, revealing the presence of neural progenitors, excitatory and inhibitory neurons, astrocytes and oligodendrocyte precursor cells, and photosensitive cells (Quadrato et al., 2017; Kanton et al., 2019, 2020; Tanaka et al., 2020). Despite this high cellular and spatial complexity offers the opportunity to model the interactions between different brain regions, the stochastic nature of spontaneous neural differentiation often results in high variability across batches of differentiated organoids that makes quantitative studies challenging. Many modifications of this pioneer protocol have been devised in order to guide the development of more reproducible region-specific organoids or to control different organoid features through bioengineering approaches.

Guided strategies direct regional fate specification by using patterning factors such as morphogens and small molecules (Eiraku et al., 2008; Danjo et al., 2011; Kadoshima et al., 2013). Indeed, self-patterning of organoids precursors can be restricted to forebrain fate by inhibiting TGF-β, BMP, and WNT pathways (Figure 1A; Eiraku et al., 2008; Kadoshima et al., 2013). By supplying additional patterning factors (such as WNT3A, SHH, insulin, and BMP7), forebrain fate can be further confined to produce organoids resembling any discrete part of the forebrain region, including cerebral cortex, optic cup, hippocampal, choroid plexus, subpallium, thalamus, and hypothalamus (Wataya et al., 2008; Eiraku et al., 2011; Nakano et al., 2012; Germain et al., 2013; Kadoshima et al., 2013; Liu et al., 2013; Maroof et al., 2013; Nicholas et al., 2013; Mariani et al., 2015; Sakaguchi et al., 2015; Qian et al., 2016; Shiraishi et al., 2017; Kim et al., 2019b; Xiang et al., 2020a). Midbrain organoids are generated by combining TGF-β and BMP inhibition with WNT and SHH activation, and FGF8 treatment (Jo et al., 2016; Kim et al., 2019a), whereas cerebellum organoids are produced by timed and combinatory treatment with a series of patterning factors, including TGF-β and BMP inhibitors, insulin, FGF2, FGF19, and SDF1 (Figure 1A; Muguruma et al., 2015).

Interestingly, regionally specified cerebral organoids can be fused to each other to model the interaction between distinct brain regions and to assess fundamental features of brain development and disease, such as cell migration and long-range connectivity (Figure 1B; Bagley et al., 2017; Birey et al., 2017; Mich et al., 2017; Xiang et al., 2017, 2018, 2019). For example, dorsal and ventral forebrain organoids can be assembled *in vitro* to recapitulate the typical saltatory migration and integration into cortical circuits of interneurons observed in the fetal brain (Birey et al., 2017). Instead, the creation of reciprocal thalamocortical axon projections, which establishment is critically involved in sensory-motor processing and attention, can be modeled *in vitro* by fusing thalamic and cortical organoids (Xiang et al., 2019).

A series of advances in brain organoids technology have been achieved by integrating organoids culture with bioengineering methodologies, particularly biomaterials, microfabricated, and microfluidic devices, in order to enable a better spatiotemporal control of cellular differentiation and organoid morphogenesis (Figure 1C). Indeed, inceptive hPSC aggregates and embryoid bodies dimension and morphology can be easily standardized by using micropatterned substrates, micropillar array or microwells, increasing brain organoid generation reliability, and reproducibility (Knight et al., 2015; Zhu et al., 2017; Haremaki et al., 2019; Velasco et al., 2019; Yoon et al., 2019). Alternatively, fiber microfilaments have been used as a floating scaffold to generate elongated embryoid bodies (Lancaster et al., 2017). The resulting microfilament engineered cerebral organoids (enCORs) were characterized by enhanced neuroectoderm formation and improved cortical development (Lancaster et al., 2017). Others efforts to better control the morphology of developing organoids include the employ of hydrogels or microfabricated culture chambers with defined microscale dimensions (Karzbrun et al., 2018; McNulty et al., 2019). Carefully designed microfluidic devices could be useful to generate morphogen gradients in order to guide organoids patterning (Manfrin et al., 2019).

Additional fundamental aspects of recent advances in brain organoids technology include attempts to incorporate non-neuronal components, such as microglia and vasculature, that are of primary importance in brain development and disease (Figures 1D,E; Obermeier et al., 2013; Beggs and Salter, 2016).

Cells with typical microglial morphology, molecular phenotype and function can innately develop within unguided and self-patterned cerebral organoids, as demonstrated by Ormel et al. (2018). Otherwise, microglial component can be integrated in region-specific organoids by co-culture with human induced PSC-derived microglial-like cells (Song et al., 2019).

As depicted in Figure 1E, several strategies have been explored also to achieve cerebral organoids vascularization. Indeed, formation of a mature vascular system is essential to sustain organoids growth capacity during long-term cultures and to better recapitulate *in vitro* the complex processes happening *in vivo*, and involving the simultaneous and integrated development of organs and vasculature. Vascularized cerebral organoids have been initially obtained by organoids transplantation into the brain of immunodeficient mice (Mansour et al., 2018) or by co-culture with endothelial cells or their progenitors during organoids formation (Pham et al., 2018). More recently, cerebral organoids have been co-differentiated with endothelial-like cells by supplementation of VEGF during organoids derivation (Ham et al., 2019) or by targeted overexpression of the transcription factor hETV2 in a small subset of initial hPSC population (Cakir et al., 2019). In both cases, a functional vascular-like system appeared without reducing neural morphogenesis, rather favoring a better organoid maturation (Cakir et al., 2019).

## Brain Organoids in Autism Spectrum Disorders

Autism spectrum disorders are defined as a combination of neurodevelopmental diseases (Hodges et al., 2020). Symptoms are characterized by lack of development of social and emotional relationships, difficulties in the use of language, apathy, rigidity in movements and repetitive and maniac behaviors. So far, it has been hard to well define a diagnosis because ASD marks are often associated with other psychiatric features as well as ID, epilepsy, and attention-deficit hyperactivity disorder (Moreno-De-Luca et al., 2013). Genetic variants and environmental factors are known to play important roles in the pathogenesis of ASD (Buxbaum et al., 2007). For instance, genetic variants involving synaptic genes are emerging as recurrent causative factors in the etiology of ASDs (Südhof, 2008; Zoghbi and Bear, 2012; Salpietro et al., 2019). Moreover, different authors speculate on the role of the unbalance between excitatory and inhibitory circuits in the pathogenesis of these disorders (Casanova et al., 2003; Rubenstein, 2010). Recently, genomic data and gene networks analysis suggested a common cause for ASDs during the embryonic development of the cerebral cortex (Parikshak et al., 2013; Willsey et al.,2013). In this context, 3D *in vitro* models are increasing our capability to study neuropsychiatric diseases (Yang and Shcheglovitov, 2020).

**FIGURE 2:**
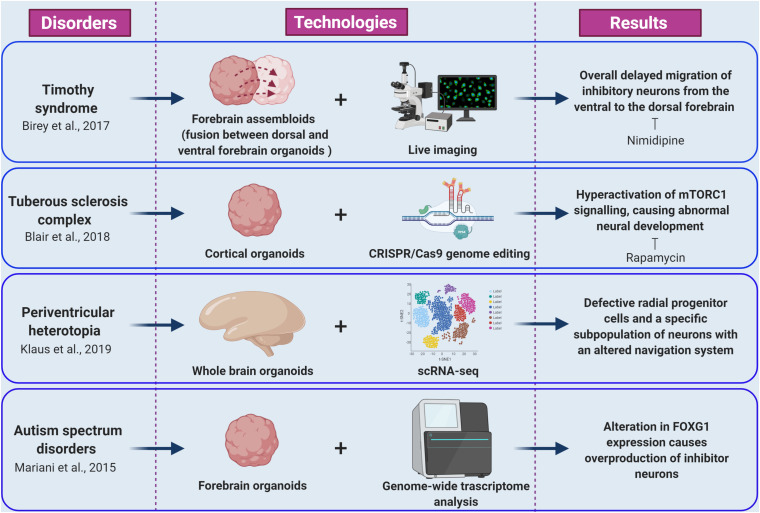
Cellular and molecular phenotypes revealed by brain organoid models of epilepsy and ASDs. The figure summarize how integration of brain organoids technology (forebrain assembloids, cortical organoids, whole brain organoids, and forebrain organoids) with multiple experimental approaches (including live imaging, CRISPR/Cas9 genome editing, scRNA-seq, and genome wide transcriptome analysis) allowed the understanding of various molecular mechanisms underlying epileptic and autism spectrum disorders (created with biorender.com).

As a first example, Mariani and co-workers studied the early cortical development in patients with idiopathic ASD characterized by increased head/brain size (macrocephaly), that is one of the most common sign in ASDs phenotypes (Courchesne et al., 2001; Mariani et al., 2015). Transcriptomic and cellular studies on iPSC-derived forebrain organoids from autism patients revealed alterations in cell-cycle and in synaptic growth (Figure 2). Notably, ASD organoids showed an increased production of inhibitory neurons caused by increased expression of the transcription factor *FOXG1*, highlighting the role of this gene as a molecular signature of idiopathic ASD and a potential therapeutic target (Mariani et al., 2015; Hou et al., 2020).

Growing knowledge on ASD risk variants and on gene networks involved at different time points and in different cell types during neurodevelopment process suggest that ASD pathologies may arise from combined deficiencies during early stages of cortical development (Parikshak et al., 2013; Willsey et al., 2013). Indeed, Schafer et al. (2019) by combining transcriptomic analysis, cerebral organoids generation and direct iPSCs-to-neuron conversion demonstrated how autism-related neurodevelopmental alterations are triggered by temporal dysregulation of specific gene networks and morphological growth acceleration occurring during early neural development. Interestingly, while ASD alterations were recapitulated on forebrain organoids generated from idiopathic ASD patients-derived iPSCs, the direct conversion of ASD iPSCs to mature neurons abolished ASD-associated phenotypes (Schafer et al., 2019). These findings indicate that some ASD-associated anomalies are likely to be the consequence of pathological events triggered during neural stem cells stages, and possibly involving epigenetic changes (Schafer et al., 2019).

## Brain Organoids and Epilepsy

Timothy syndrome (TS) represents a successful application of brain organoids technology to the investigation of human brain development disorders and epilepsy. This disorder is caused by mutations in *CACNA1C*, the gene encoding the L-type calcium channel Cav1.2 α subunit, producing abnormal inhibitory neurons. The generation of cerebral organoids derived from TS-iPSC, gained insight to inhibitory neurons migration pattern during brain development (Birey et al., 2017). Indeed, live imaging of TS-forebrain assembloids (fusion between cerebral organoids resembling dorsal and ventral forebrain) revealed a cell-autonomous defect consisting in overall delayed migration of inhibitory neurons from the ventral to the dorsal forebrain (Figure 2; Birey et al., 2017). Importantly, this phenotype was rescued by blocking the activity of L-type calcium channels by nimodipine (Birey et al., 2017). Importantly, this study was the first example of fused organoid approach to *in vitro* modeling of neuronal circuits involving distinct brain regions (Birey et al., 2017; Koo et al., 2019).

Given the limited access to subjects with rare epilepsy mutations, brain organoid technology combined with CRISPR/Cas9 genome editing methods can be particularly useful to investigate the effects of rare patient-specific variants in an isogenic control background.

Tuberous sclerosis complex (TSC) disease is an autosomal dominant disorder associated with epilepsy, ID, and benign tumors of the brain, heart, skin, and kidney. TSC is caused by mutations in the *TSC1* or *TSC2* genes. The proteins encoded by these genes form a heterodimeric complex that negatively regulates mechanistic target of rapamycin complex 1 (mTORC1) signaling (Saxton and Sabatini, 2017). The origins of the neurological aspects of TSC are largely unknown. However, patients present with characteristic malformations, called cortical tubers, which are macroscopic regions of disorganized and dysmorphic cells in the cortex (Crino, 2013). The proposed model of cortical tuber formation is that somatic “second-hit” mutations in patients with heterozygous germ line mutations result in loss of function of the TSC1-TSC2 complex and hyperactivation of mTORC1 signaling in a subset of cortical progenitor cells (Magri and Galli, 2013; Crino, 2016). Recently, Blair and colleagues combined brain organoid and CRISPR/Cas9 gene editing methods as a means to investigate the “two-hit” hypothesis of cortical tuber development (Figure 2; Blair et al., 2018; Saber and Sahin, 2020). Homozygous, but not heterozygous, loss of *TSC1* or *TSC2* disrupts the developmental suppression of mTORC1 signaling providing strong support for the two-hit hypothesis of TSC pathophysiology. They also showed that early rapamycin treatment of the brain organoids prevented development of the abnormal phenotypes (Blair et al., 2018; Niu and Parent, 2019).

Lately, iPSCs from patients carrying mutations in *DCSH1* and *FAT4* genes were used to model periventricular heterotopia (PH), a disorder of neuronal migration, using self-patterned brain organoids (Klaus et al., 2019). While analyzing the impact of the *DCSH1* and *FAT4* mutations on organoids generation, the authors found defective radial progenitor cells, which should guide the neurons to the correct final destination (Figure 2). In addition, scRNA-seq analysis revealed a specific subpopulation of neurons with an altered navigation system, which changed their migratory dynamics and led to compromised equipment for synaptic signaling (Klaus et al., 2019).

Angelman syndrome (AS) is a debilitating neurological disorder caused by mutation of the E3A ubiquitin protein ligase (*UBE3A*) gene. Although, AS mice showed impaired synaptic connectivity and altered plasticity associated with abnormal behavior, the pathological mechanisms underlying epilepsy, as well as the biological substrate of UBE3A, remained unknown. Recently, by using brain organoids, Sun et al. (2019) demonstrated that UBE3A suppresses neuronal hyperexcitability via ubiquitin-mediated degradation of calcium and voltage-dependent big potassium (BK) channels. Consequently, neuronal excitability can be normalized and seizure susceptibility ameliorated by antagonizing BK channels (Sun et al., 2019).

Rett syndrome and Bosch-Boonstra-Schaaf optic atrophy syndrome (BBSOAS) are two additional examples of recent studies in which brain organoids have been successfully used. Rett syndrome (RTT) is a severe X-linked dominant NDD affecting females caused primarily by mutations in *MECP2*, which encodes a multifunctional epigenetic regulator. Mellios et al. (2018) used *MECP2*-deficient and patient-derived cerebral organoids to identify defects in neurogenesis, neuronal differentiation, and migration. Xiang et al. (2020b) revealed cell-type-specific transcriptome impairment in *MeCP2* mutant region-specific brain organoids.

Bosch-Boonstra-Schaaf optic atrophy syndrome is a recently described autosomal dominant disorder, caused by loss-of-function mutations of the transcriptional regulator *NR2F1*. Cerebral organoids with reduced NR2F1 levels displayed altered neurogenesis and expression of *PAX6*, a gene that has a crucial role in neurogenesis in the developing cortex (Bertacchi et al., 2020).

## Conclusion and Perspectives

Brain organoids technology represent a tremendous breakthrough for the study of brain development, function, evolution and disorders and is still in his infancy. Continuous advances of 3D-culture systems and their integration with state-of-the-art biotechnology and bioengineering approaches, including single-cell transcriptomics, genome-editing, cell reprogramming and biomaterials, will lead the cerebral organoids to probably become the best *in vitro* model system for neurodevelopment studies in humans. Particularly, further advances will be necessary to standardize organoids generation protocols in order to increase their reproducibility and, hopefully, to reduce their costs. The development of new experimental procedures and functional assays will be important to explore the brain organoids ability to mimic even more complex processes involved in human neurodevelopment in health and diseases, and, therefore, to increase the translational value of this technology and its relevance in clinical applications and precision medicine approaches.

## Author Contributions

SB, IM, VS, and PS wrote the manuscript and prepared the figures. MI, VS, FZ, and PS revised the manuscript. VS and PS coordinated the preparation of the review. All authors contributed to the article and approved the submitted version.

## Conflict of Interest

The authors declare that the research was conducted in the absence of any commercial or financial relationships that could be construed as a potential conflict of interest.
